# Quality of life of Syrian refugees living in camps in the Kurdistan Region of Iraq

**DOI:** 10.7717/peerj.670

**Published:** 2014-11-11

**Authors:** Izaddin A. Aziz, Claire V. Hutchinson, John Maltby

**Affiliations:** University of Leicester, Leicester, United Kingdom

**Keywords:** Refugees, Quality of life, Psychological health, Physical health, Syria

## Abstract

The current study explores the perceived quality of life of Syrian refugees who have entered the Kurdistan Region of Iraq. Two hundred and seventy participants residing in refugee camps in the Erbil region in Kurdistan completed the WHOQOL-BREF, which measures Quality of Life (QOL) within four domains; physical, psychological, social relationships and environment. Syrian refugees in Kurdistan scored significantly lower for general population norms on physical health, psychological and environment QOL, and score significantly lower for physical health and psychological QOL for refugees in the Gaza strip. However, respondents in the current sample scored significantly higher on environment QOL compared to refugees in the Gaza strip, and significantly higher on all the QOL domains than those reported for refugees in West Africa. Finally, Syrian refugees in Kurdistan scored significantly higher than general population norms for social relationships QOL. The current findings provide the first report of QOL domain scores among Syrian refugees in the Kurdistan camps and suggest that social relationships and environmental QOL circumstances are relatively satisfactory, and that further investigation might be focused on physical and psychological QOL.

## Introduction

The war in Syria has led to the worst humanitarian crisis of the 21st century. According to United Nations Refugee Agency figures, over 2.5 million people have fled the Syrian conflict, entering as refugees neighboring countries of Turkey, Egypt, Lebanon, Jordan and Iraq. The United Nations Refugee Agency (UNHCR) recorded that by the end of July 2012, 9,503 Syrians had registered as refugees in Iraq who have left Syria for a number of political, economic and social reasons. By the end of February 2013, this number had increased over 10-fold to 102,447 (UNHCR, 2013). By February 2014, the figure stood at 225,548 (UNHCR, 2014a) and continues to increase. As of 5th March 2014, 226,934 people had registered as refugees in Iraq. The majority (around 97%) are registered in the Kurdistan Region in Northern Iraq, in and around the cities of Duhok (109,979 registered refugees), Erbil (84,881 registered refugees) and Suleimaniyah (25,134) (UNHCR, 2014b). Around 60% of Syrian refugees are hosted within communities across Kurdistan and the remaining 40% live in refugee camps (UNHCR, 2014a). When Syrian refugees first began arriving in 2012, most registered in the Directorate of Duhok, near the Peshkhabour border with Syria. This led to the opening of the Domiz camp on 01 April 2012. It remains the largest permanent camp with a population of 58,500, as of 28 February 2014. In 2013, as the number of refugees seeking asylum increased, a further four permanent camps were opened in the Directorate of Erbil: Kawergosk (15 August 2013), Qushtapa (19 August 2013), Basirma (26 August 2013) and Darashakran (29 Sept 2013), with a combined population of 28,208, as of 28th February 2014 (United Nations Refugee Agency Information Management Unit, 2014).

International Aid Agencies are working in collaboration with The Kurdistan Regional Government (KRG) to provide shelter, food, water, healthcare, education and employment for Syrian refugees (UNHCR, 2014c). However, given the sheer numbers of people in need, it is an extremely, and increasingly, difficult situation to manage. Attending, for example, to the complex healthcare needs of such a large population represents a major challenge. The UN Refugee Agency records information from refugees about their physical health complaints and clinical mental health problems at the point of registration but this is almost impossible to monitor on a follow-up basis, given the many challenges and constraints posed by the current crisis. As a result, many psychological issues facing those who live in refugee camps are very unlikely to be addressed or detected.

Despite the profound effect of war and forced migration on people’s living conditions, surprisingly little attention has been paid to the psychological impact of being a refugee. Studies that have investigated this issue have found that the prevalence of psychological illness is relatively high in refugee groups (Gerritsen et al., 2006). Research suggests that poor perceived *present* Quality of Life (QOL) may be the most significant factor in psychological illness and stress related disorders in refugee populations (Akinyemi et al., 2012; Carlsson, Olsen & Mortensen, 2006; Fazel, Wheeler & Danesh, 2005; Matanov et al., 2013; Tang & Fox, 2001). These findings support the World Health Organization’s position concerning the importance of subjective quality of life as a measure of how an individual perceives “their position in life in the context of the culture and value systems in which they live and in relation to their goals, expectations, standards and concerns” (p. 1, World Health Organization, 1997).

At present there is, at least to our knowledge, no data concerning the known perceived QOL of Syrian refugees who have entered the Kurdistan Region of Iraq. These are important issues that, given the sheer scale of the Syrian refugee crisis, have fundamental implications for the future health and well-being of a large number of people. In the present study, we report on the World Health Organization Quality of Life Assessment (WHOQOL-BREF) scores among Syrian refugees living in refugee camps in The Directorate of Erbil, Iraqi-Kurdistan. To provide context to our findings we compare WHOQOL-BREF scores among the current sample to other reports of WHOQOL-BREF scores among other refugee reports.

## Method

### Sample

Two hundred and seventy Kurdish nationalist refugees (135 males, 135 females), aged 18 to 60 (*M* = 29.26 years, SD = 9.7) from Syria, residing in refugee camps located in Kurdistan took part in the study. The sample used in this study was residing in the Erbil Governorate camps located on four sites: Qushtpa (*n* = 67), Kawrgosk (*n* = 67), Basirma (*n* = 68) and Darashakran (*n* = 68) in January 2014. At each site, the researchers split the map of the site into four zones, with the aim of obtaining around 20 respondents from each zone. In terms of selection and inclusion criteria, the researcher selected the second residence from each alley (moving on to the next residence in that alley if an interview was declined). One member from each family was chosen, who had to be 18 years old or over, and did not have special needs considerations. Equal numbers of respondents were sought from each gender, and to avoid selection bias on the part of the researcher, where there were multiple candidates in any residence for the interview, the individual with the closest birthday to the interview date was chosen.

Of these respondents, the most dominant demographic statistics were that 42.6% of respondents reported highest qualification was having completed secondary education (31.5% of respondents had completed tertiary education, and 17.4% of respondents had completed a primary education) and 58.1% of respondents reported being married (with the next highest frequency being that 40.5% of respondents were single and 1.9% of respondents were separated).

### Measures

The WHOQOL-BREF is the short 26-item form of the larger WHOQOL-100 assessment (The WHOQOL Group, 1995) that yields four QOL domains: physical health (7 items; e.g., “How much do you need medical treatment to function in your daily life?”), psychological QOL (6 items; e.g., “To what extent do you feel life to be meaningful?”), social QOL (3 items; e.g., “How satisfied are you with your personal relationships?”), and environmental QOL (8 items; e.g., “How safe do you feel in your daily life?”). Responses are scored via five-point response scales with various anchor statements (e.g., from 1 [*Very dissatisfied*]  or  [*Very poor*] to 5 [*Very satisfied*] or [*Very good*]). The WHOQOL-BREF can be scored in three ways; through raw scores and two transformation methods; the first that creates domain scores within the range of 4–20, and the second that creates domain scores within the range of 0–100.

The WHOQOL-BREF’s psychometric properties have been analyzed using cross-sectional data from 11,830 adults from 23 countries (Skevington, Lofty & O’Connell, 2004) and is a valid assessment across cultures and socioeconomic status (Hawthorne, Herrman & Murphy, 2006; Skevington, Lofty & O’Connell, 2004). Most Syrian refugees in these camps tend to speak the Kurdish language, but have different dialects from the Iraqi Kurdish. However, they are also able to speak the Arabic language. Therefore they were given the Arabic version of the *World Health Organization Quality of Life Scale—Brief* (WHOQOL-BREF) (Skevington, Lofty & O’Connell, 2004; The WHOQOL Group, 1998). The reliability and validity of Arabic versions of the WHOQOL-BREF have been demonstrated among large Arabic-speaking samples (Ohaeri & Awadalla, 2009). On this occasion, we removed one of the social relationships QOL items (“How satisfied are you with your sex life?”) due to concerns over the respondents’ potential sensitivity to the question. According to the WHOQOL-BREF manual the transformational methods for scoring of the scale allows for missing items.

### Ethics

The study received ethical approval from the University of Leicester’s School of Psychology Ethics Board whose ethical procedures conform to those of the British Psychological Society (http://www.bps.org.uk/sites/default/files/documents/code_of_human_research_ethics.pdf). The Ethics Reference for the Ethics Board was jm148-851fa. All participants were 18 years of age or over and provided free and informed consent to take part in the study. Formal procedures and permission to visit the camps were given by the General Director of Academic Missions and Cultural Relations and the Democracy and Human Rights Research Institute.

## Results

We found two reports of mean statistics for scores on the WHOQOL-BREF from refugee samples that were not from a clinical population. These reports were from samples from refugee populations residing in West Africa (Akinyemi et al., 2012) and the Gaza Strip (Eljed et al., 2006). Together, with the overall norm data for the WHOQOL-BREF from 11,830 adults from 23 countries (Skevington, Lofty & O’Connell, 2004), these three reports provided information to facilitate statistical mean score comparisons between the current sample and three other samples.

Table 1 shows a set of mean comparisons between Syrian refugees in Kurdistan and overall norm data for the WHOQOL-BREF. This comparison uses transformed domain scores within a range of 4–20. As our sample data has a missing item, we recomputed the mean/SD score for social relationships QOL for the general population norm data using the frequency responses that have been provided for these two social relationship items (Skevington, Lofty & O’Connell, 2004). In this table, we also provide effect sizes for the comparisons computed for unequal sample sizes, for which *d* ≥ .8 represents a large effect size, .5 ≤ *d* < .8 represents a moderate effect size, and .2 ≤ *d* < .5 represents a small effect size (Cohen, 1988). In terms of the comparison with the norm data, the refugees residing in Kurdistan scored significantly lower on physical health, psychology and environment QOL, but significantly higher on social relationships QOL. In terms of effect size, the differences for physical health and psychological QOL are of a large effect size, the differences for the environment QOL are of a moderate effect size, but the difference reported for social relationships QOL does not even meet the criteria of a small effect size.

**Table 1:** Mean (SD) score comparisons for WHOQOL-BREF domain scores (range 4–20) between Kurdistan refugees and adults across 23 countries (*n* = 11,830) from Skevington, Lofty & O’Connell (2004).

	Syrian Refugees in Kurdistan (*n* = 270)		Adults across 23 countries (*n* = 11, 830)			
	Transformed Scores (4–20)					
	Mean	SD	Mean	SD	*t*	*d*
Physical health	13.26	2.45	16.2	2.9	−19.41^**^	1.20
Psychological	12.62	2.45	15.0	2.8	−15.73^**^	0.97
Social relationships	15.23	2.82	14.7 (14.3)^a^	3.7 (3.2)	2.99^*^	0.18
Environment	11.66	2.39	13.5	2.6	−12.48^**^	0.77

**Notes.**

* *p* < .01.

** *p* < .001.

a Original mean (SD) scores provided by Skevington, Lofty & O’Connell (2004) in brackets.

Table 2 shows the comparison with the first of the two refugee samples, refugees resident in West Africa (Akinyemi et al., 2012). For this sample, mean scores were presented as raw scores. Therefore, we have presented mean scores for the Syrian refugees in accordance with this. Across all domains of the QOL scale, the refugees residing in Kurdistan scored significantly higher than those reported in West Africa, with these differences ranging from a moderate effect size (social relationships QOL) to a large effect size (physical health, psychological and environment QOL).

**Table 2:** Mean (SD) score comparisons for WHOQOL-BREF raw scores between Syrian refugees residing in Kurdistan and refugees residing in West Africa.

	Refugees in Kurdistan (*n* = 270)		Refugees in West Africa Akinyemi et al. (2012) (*n* = 444)			
	Raw scores					
	Mean	SD	Mean	SD	*t*	*d*
Physical health	23.21	4.29	19.45	4.18	11.47^*^	0.89
Psychological	20.30	3.62	16.86	4.04	11.78^*^	0.91
Social relationships	10.10^a^	2.25	8.66	2.59	7.83^*^	0.60
Environment	22.85	4.62	18.88	5.03	10.76^*^	0.83

**Notes.**

* *p* < .001.

a Raw score for 2 items is weighted for comparison against a 3 item score.

Table 3 shows a comparison with a second refugee sample, residents in the Gaza Strip (Eljed et al., 2006). For this study, mean scores were presented as transformed domain scores with a range of 0–100. Therefore, we have presented the mean scores for the Syrian refugees in accordance with this. For the physical and psychological domains, the refugees residing in Kurdistan scored significantly lower than for those refugees in the Gaza Strip, with these differences being of a large effect size. For the environment QOL domain refugees residing in Kurdistan scored higher than for those refugees in the Gaza Strip with this difference being of a moderate effect size. No significant difference was found between Syrian refugees in Kurdistan and refugees residing in the Gaza strip for the social relationships QOL.

**Table 3:** Mean (SD) score comparisons for WHOQOL-BREF transformed scores (0–100) between Syrian refugees residing in Kurdistan and refugees residing in the Gaza strip.

	Refugees in Kurdistan (*n* = 270)		Refugees in the Gaza strip Eljed et al. (2006) (*n* = 197)			
	Transformed scores (0–100)					
	Mean	SD	Mean	SD	*t*	*d*
Physical health	58.12	15.45	75.9	20.92	−9.31^*^	0.87
Psychological	53.82	15.35	70.0	21.65	−9.02^*^	0.85
Social relationships	70.41	17.61	71.4	19.48	−0.57	0.05
Environment	46.58	15.15	36.2	20.20	6.11^*^	0.57

**Notes.**

* *p* < .001.

## Discussion

The current findings suggest the QOL among Syrian refugees in Kurdistan falls largely within a range of QOL scores that have been reported from other samples. In summary, the scores among the current sample are: (a) statistically significantly lower than general population norms (Skevington, Lofty & O’Connell, 2004) for three QOL domains (physical, psychological and environment) and Gaza Strip refugees (Eljed et al., 2006) on two QOL domains (physical and psychological); and (b) statistically significantly higher than West Africa refugees (Akinyemi et al., 2012) on all the QOL domains, Gaza Strip refugees on one QOL domain (environment), and general population norms on one domain (social relationships).

A key finding from these comparisons is that Syrian refugees score higher than general population norms on social relationships QOL. At face value, this suggests that Syrian refugees report being satisfied with their personal relationships and friendships more than those in the general population. The explanation for this difference may be that the current sample comprises many individuals who have moved away from the conflict in Syria with their family and friends. Therefore, the higher scores reflect a shared experience and purposeful support within a current social network that has been heightened in the context of the current conflict, whereas this level of social support which would not necessarily be present for many members of a general population as it would not be required. It is worth noting that the statistical effect size of this higher score is negligible (a magnitude that is less than small [*d* = .2]) and therefore any statistical significant difference could be attributed to sample size. Moreover, there may be a concern about making the comparison while omitting of one of the items from the scale, although the scoring of the WHOQOL-BREF allows for the omission of items, and we have made a comparable alteration to the population mean scores. Notwithstanding those caveats, the current findings suggest that, even if the statistically significant higher difference is not robust, the social relationships QOL scores compare favorably (by not being statistically significantly lower as with the other QOL domains) to the population mean.

Another key finding is that Syrian refugees scores higher on environment QOL than the other two refugee samples. This suggests that those respondents in the Kurdistan camps are relatively more satisfied with the living conditions, and access to health and transport services to respondents from the other two refugee camps at the time of those surveys. Therefore, it seems that the environmental provisions made by the United Nations Refugee Agency in the camp could be viewed as favorable.

The findings seem to invite special attention for further research among Syrian refugees in terms of the physical and psychological QOL domains, largely because these scores are lower to a large effect size than those means reported among Gaza Strip refugees. It is likely that the recency of the conflict in Syria and movement to the refugee camps has led to heightened levels of health and psychological problems, however, as lower physical and psychological QOL can be indicative of stress-related disorders (Carlsson, Olsen & Mortensen, 2006; Fazel, Wheeler & Danesh, 2005) this requires further investigation. Therefore this finding indicates that if there is current concern for policy makers or researchers, there may be a need to prioritize aspects of physical and psychological QOL among Syrian refugees.

Despite these speculations, it is important that seeking any exacting social or policy analysis in these comparisons is mostly redundant to the immeasurable variance in the nations, context and time periods considered. However, the current findings do provide some key indicators to QOL among Syrian refugees in Kurdistan, in terms of comparisons to the general population and other refugee camps. Namely social relationships and environmental QOL circumstances are relatively satisfactory, but if further investigation is needed, then a key focus might be the consideration of physical and psychological QOL.

## Supplemental Information

10.7717/peerj.670/supp-1 Supplemental Information 1 Datafile
